# T-regs/Th17 function defect in systemic autoimmunity as a result of "recent thymic emigrants" maturation defect

**DOI:** 10.1186/ar3622

**Published:** 2012-02-09

**Authors:** Mark Goloviznin, Natalia Lakhonina, Alexander Yarilin, Yulia Buldakova, Vitaly Timofeev, Tatiana Kremenchugskaya, Marina Struchkova

**Affiliations:** 1 Department of Internal Diseases of Dental Faculty, Moscow State University of Medicine and Dentistry, Russia; 2 Laboratory of Cell Immunology, Research Center Institute of Immunology, Moscow, Russia; 3 Department of Faculty Therapy of Russian State Medical University, Moscow, Russia

## 

T-regs and Th17 cells are the new generation of CD4+T-cells which play crucial role in autoimmunity. Both of subsets can influence each other and probably have common precursor. A key question for understanding the mechanism of autoimmunity is to recognize how T-regs and Th17 cells turn from self-protection to autoreactivity. Based on literature data and own observations, we have constructed a conception of age-dependent thymic T-cells maturation "peripherialisation" as cause of errors in Th17-T-reg cells interrelations. The connection of T-regs with thymus is determined currently. Connection of Th17 cells with thymus remains to be determined properly. Main, there may be naturally occurring Tregs of thymic origin that are resistant to cell death and serve as "reserve pool" for autoimmunity protective suppressors. This mechanism could be affected by external factors producing profound lymphopenia [1]. Previously we found that RA patients with numerous rheumatoid nodules (RN) and lymphopenia had statistically reliable decrease of CD3+T-cells level. We found definite negative correlation between CD3+PBL amount and RN number (p = 0,029). In all RA patients with and without RN we didn't found the decrease of CD4 receptor. Hereby we expected to find unusual CD3-4+ and CD3-8+ cells in RA. Otherwise the percentage of CD3+4+ and CD3+8+ cells was normal in general. But in 4 RA patients after magnetic separation of CD3+T-cells we detected reliable amount of CD3-4+ lymphocytes (25-28%) These cells were not detected before separation. One of possible explanation of this phenomenon is CD3 molecule modulation after the contact with anti-CD3 antibodies conjugated with magnetic particles. So the presence of T-cells with unusual phenotype in peripheral blood of RA patients doesn't give absolute evidence of T-cells maturation disorders. According to our viewpoint "recent thymic emigrants" (RTE) fraction presence among T-regs and hypothetically among Th17-cells is the sign of normal Th17/T-regs function. Otherwise the absence of RTE among them leads to immunopathology. CD31 receptor and T cell receptor rearrangement excision circles (TREC) are now markers of RTE. We investigated the number of CD4+CD31+T-cells in RA patients. The preliminary results permit us to suggest the diminution of RTE in RA (less then 1%/ml) We also found the diminution of TREC amount in PBL of 22 rheumatoid arthritis patients, (Median 0,035539 units). FOXP3, RORγ, RORα and CD31 expression in RA will permit to establish role of RTE in autoimmunity.
